# Supplementary material for: A systematic review of Hepatitis B virus (HBV) prevalence and genotypes in Kenya: Data to inform clinical care and health policy
Source: PLOS Glob Public Health. 2023 Jan 31;3(1):e0001165. doi: 10.1371/journal.pgph.0001165 (PMC10022289; doi:10.1371/journal.pgph.0001165)
Supplement: Attachment — Submitted filename: 221105 Response to reviewers.pdf [file pgph.0001165.s004.pdf]

Manuscript ref PGPH-D-22-00830_R2

Thank you to reviewer 2 and the editorial team for providing further feedback. We have provided a point-by-point response to the reviewer, and uploaded a revised manuscript (clean and tracked versions).

Reviewer #2: The revised manuscript is better than the original submission.

Thank you for positive feedback.

However, I disagree with the authors on the following points, and still believe that the authors should:

a. The studies should not be divided into groups such as low risk, moderate risk, high risk and very high risk for HBV infection. Even the authors' results show prevalence to be similar in moderate and high risk groups. Therefore, the studies should instead be grouped as: (i) those in groups likely to represent general population (healthy persons, antenatal women, etc -- the current low risk group), (ii) people living with HIV (the current moderate risk group), (iii) IVDUs and (iv)MSM (the last 2 are currently in high-risk group).

As previously discussed, we grouped the studies prior to undertaking the full analysis, and would prefer to stick to the analysis protocol determined *a priori* rather than changing our methods after seeing the results. However, based on this latest feedback, we did review the suggestion of dividing the groups further. Dividing the high-risk group into IVDU and MSM leaves only 2 studies in each group, and then one further study in female sex workers that is unclassified. We think the number of studies in each group is therefore too small to be meaningful and leaves an 'orphan' study that is not classified. Thus on the grounds of (i) adhering to our pre-analysis protocol and (ii) retaining sufficient data in each group, we have elected not to make further changes.

In the methods section headed '**Study heterogeneity and HBV risk groups'**, we have added further justification for these groupings; the modified text now reads as follows: 'On the grounds of significant heterogeneity in the populations represented, we divided studies a priori into four groups representing populations with differing risks of testing positive for HBV infection. The low-risk group included studies likely to be most representative of the general population (antenatal women, healthcare workers, blood donors and the national survey). The moderate risk group consisted of studies containing populations living with HIV. High-risk groups were defined as people with risk factors for acquisition of blood-borne virus infection, including people who inject drugs, men who have sex with men (MSM) and sex workers.

We have also added to the discussion section to pick up on these points – please see further details in response to point (b).

b. Calling studies in 'liver disease patients' as 'high risk' group is wrong. It is not that liver

disease is leading to a high risk of liver disease. Instead it is HBV infection which is the cause of liver disease. Hence, data on liver disease patients should not be included in the main table, and should be presented separately.

The 'risk profile' corresponds to the risk of people within that group having HBV infection (not the risk of them having liver disease). A population presenting to hospital with established liver disease in Kenya has a higher risk of testing positive for HBV infection than the general population. We have reworded the description of the risk groups in the methods as follows: 'Those presenting to hospital with hepatitis or jaundice were defined as a very high-risk group, as HBV infection is enriched in populations presenting with established liver disease, particularly if the background population has medium or high HBV prevalence.'

We have also reworded the results to add clarity; this now reads: 'three studies in people presenting to clinical services with established liver disease (defined here as very high-risk for HBV infection; total number of individuals = 492).'

Points (a) and (b) here are also picked up in the discussion, where we have rephrased the first paragraph to add clarity, and also acknowledge that the underlying risk factors for HBV infection in the 'very high risk' group are not known. The revised text reads as follows:

'In our 'low-risk' category, intended to provide estimates most reflective of the general population, the pooled prevalence estimate for HBV infection was 3.4%. Point-prevalence estimates of ~6% were obtained for the groups we defined as medium and high risk, comprising people living with HIV infection and those with other identified risk factors for blood-borne virus infection. Similar prevalence estimates in the moderate- and high-risk groups was only evident after analysis. The number of studies was too low to allow for further subdivision into individual risk groups (e.g. comparing people who inject drugs, MSM, and sex workers). In the population presenting to healthcare facilities with established symptomatic liver disease (classified here as 'very high risk'), the prevalence of HBV was 29.2% (although the underlying primary risk factor(s) for HBV acquisition in this group are not established).'

We have also added a comment to state that the division of populations into these risk groups is constrained by the number of studies available, and that risks may vary between different populations: 'Our risk groups were determined *a priori* based on existing understanding of the distribution of HBV infection, but data were insufficient to disaggregate into more specific groups, and we recognise that the prevalence of HBV infection in populations at risk varies substantially by setting.'

c. Presenting data on 'studies with genetic data' is irrelevant. What matters it the genotype distribution, as is also clear from the study title ("HBV prevalence and genotypes in Kenya". Hence, the three studies that contain no useful data or data on drug resistance mutations etc should be excluded.

All 8 papers identified in this review containing genetic information undertook genotyping as described in the first sentence of ‘iv) identification of HBV sequences’ in the results section. In order to clarify this further, we have also added to the legend of Figure 1 to say ‘All eight studies included for genetic analysis contain information on HBV genotype.’ We have also expanded the footnotes for Table 2 to clarify this point: ‘Studies marked with an asterisk (*) are those which presented HBV genetic information including genotype.’ To add clarity to Table 3, where genotypes are presented, we have added to the header to say ‘Data from 8 studies marked * in Table 2’.

Therefore, we disagree with the reviewer that there are three studies that contain ‘no useful data’, and we have retained all 8 of the studies identified in this category. We believe the amendments to the text, figure and table annotations make this much more transparent.
