# Supplementary material for: A systematic review of Hepatitis B virus (HBV) prevalence and genotypes in Kenya: Data to inform clinical care and health policy
Source: PLOS Glob Public Health. 2023 Jan 31;3(1):e0001165. doi: 10.1371/journal.pgph.0001165 (PMC10022289; doi:10.1371/journal.pgph.0001165)
Supplement: Attachment — Submitted filename: 221007 Revision cover letter and response to reviews.docx [file pgph.0001165.s003.docx]

**Abraham D. Flaxman, Ph.D.**

**Academic Editor**

**PLOS Global Public Health**

**16^th^ September 2022**

Dear Editorial Office,

**Ref: PGPH-D-22-00830, A systematic review of Hepatitis B virus (HBV) prevalence and genotypes in Kenya: Data to inform clinical care and health policy. Louise Downs et al.**

Thank you for the review of our article; we are grateful for positive feedback and constructive suggestions. We are now resubmitting with changes as suggested by the Editorial Office and by two reviewers. Detailed responses to each point are included below.

Thank you for your consideration, and we will look forward to hearing back from you in due course.

Philippa Matthews (corresponding author)

Louise Downs (first author)

**Journal Requirements:**

1. Please send a completed 'Competing Interests' statement, including any COIs declared by your co-authors. If you have no competing interests to declare, please state "The authors have declared that no competing interests exist". Otherwise please declare all competing interests beginning with the statement "I have read the journal's policy and the authors of this manuscript have the following competing interests:"

**Author Response:** This has been altered in the manuscript.

2. Please insert an Ethics Statement at the beginning of your Methods section, under a subheading 'Ethics Statement'. It must include:

a. The name(s) of the Institutional Review Board(s) or Ethics Committee(s)

b. The approval number(s), or a statement that approval was granted by the named board(s)

c. (for human participants/donors) - A statement that formal consent was obtained (must state whether verbal/written) OR the reason consent was not obtained (e.g. anonymity). NOTE: If child participants, the statement must declare that formal consent was obtained from the parent/guardian.]

**Author Response:** No ethical approval was required for this study - a statement of this nature has been added to the manuscript.

3. We ask that a manuscript source file is provided at Revision. Please upload your manuscript file as a .doc, .docx, .rtf or .tex.

We have provided a .docx file.

4. Figure 2: please (a) provide a direct link to the base layer of the map (i.e., the country or region border shape) and ensure this is also included in the figure legend; and (b) provide a link to the terms of use / license information for the base layer image or shapefile. We cannot publish proprietary or copyrighted maps (e.g. Google Maps, Mapquest) and the terms of use for your map base layer must be compatible with our CC-BY 4.0 license.

Note: if you created the map in a software program like R or ArcGIS, please locate and indicate the source of the basemap shapefile onto which data has been plotted.

If your map was obtained from a copyrighted source please amend the figure so that the base map used is from an openly available source. Alternatively, please provide explicit written permission from the copyright holder granting you the right to publish the material under our CC-BY 4.0 license.

Please note that the following CC BY licenses are compatible with PLOS license: CC BY 4.0, CC BY 2.0 and CC BY 3.0, meanwhile such licenses as CC BY-ND 3.0 and others are not compatible due to additional restrictions.

If you are unsure whether you can use a map or not, please do reach out and we will be able to help you. The following websites are good examples of where you can source open access or public domain maps:

* U.S. Geological Survey (USGS) - All maps are in the public domain. (http://www.usgs.gov)

* PlaniGlobe - All maps are published under a Creative Commons license so please cite “PlaniGlobe, http://www.planiglobe.com, CC BY 2.0” in the image credit after the caption. (http://www.planiglobe.com/?lang=enl)

* Natural Earth - All maps are public domain. (http://www.naturalearthdata.com/about/terms-of-use/)

**Author Response:** Following discussion by email, we have used a map from the public domain as the base layer for this figure. The legend has been adjusted accordingly.

**Response to Reviewers**

Reviewer #1: This is a much needed review that has great potential to generate hypothesis and influence policy. Diagnosing Hepatitis B is a rather error free process and "elimination of HBV by 2030" will only be made possible by testing more people and vaccination. Quality of evidence notwithstanding, the information discussed in this paper is of great value.

**Author response:** Thank you for positive comments.

Abstract

The authors report that studies reviewed are low quality. these studies are cross sectional which in its very nature is a limiting study design. Is it possible for the abstract to have subtitles? It not clear what the objective of the study is in the abstract. The result section on the abstract is too long. the recommendation/conclusion not congruent to the study findings.. make recommendations based on the study findings of prevalence of 3% to 29% considering the very high prevalence in the high risk group.

**Author response:**

- The Journal specifically asks that the abstract is not split into subtitles, but we have revised the abstract to improve structure and clarity.
- We have added a specific study objective as the first sentence of the abstract.
- We have shortened the abstract as much as possible, while retaining results that are of primary importance to the study.
- We have taken out the sections pertaining to HBV biomarkers and patient treatment eligibility as this was not the primary aim of the review (and in line with other feedback points, this content has also been trimmed back from the main manuscript).
- We have added to the abstract to underpin improved HBV screening, treatment and prevention in order to support progress towards elimination targets. In the main manuscript, we have also enhanced our recommendations based on the findings (see the conclusion section of discussion), and have also set a wider context for intervention based on the ‘triple elimination’ agenda (programmes that tackle HIV, HBV and syphilis together).

Introduction

The table seems confusing and unnecessary. This study is meant to describe overall prevalence and genotypes. Not to describe the assessment or management

**Author response:**

- We have removed the sections on patient assessment and management from the main manuscript, as we agree this is not a primary aim of the review. However, in line with our aims of providing evidence that supports development of public health policy to drive progress towards elimination goals, we have retained a link to these data as supplementary information. This is signposted through a final brief section in the results, which points readers to this additional material that has been generated from the same dataset.

Methodology

Could benefit from better subtitles.

**Author response:**

- We have added a couple of extra subtitles to make the different method sections clear, and improved some subtitles.

Results

I found this sections rather confusing with too much data. I appreciated the availability of figures that clarified the study a lot more.

**Author response:**

We have acted on feedback from both reviewers that we had included too much information in this section, but are pleased the figures helped with clarity. We have removed the information on other biomarkers and assessment of treatment eligibility which has removed two sub-sections of the results. This shortens the paper, and makes the narrative easier to follow.

Reviewer #2: This manuscript it's a systematic review of data on prevalence and genotype distribution off hepatitis b virus in Kenya. In all, 25 studies published in English between 2000 and 2021 but included in the analysis. The studies were mostly of low quality. The prevalence studies were divided into 4 groups, namely low risk, moderate risk high risk and very high risk for HBV infection. Information on viral genotype was available in 8 studies. Though there was enough justification for undertaking this study, the design and data have several shortcomings that limit the applicability of its results. Some of these are listed below.

**Author response:** we completely agree that many of the studies included have limitations in quality or quantity of methods and/or results, and thus we have been cautious about overall interpretation and generalisability. However, the limited amount of available data should not impact negatively on our ability to assimilate and interpret it, on the grounds that we are also careful to highlight caveats and areas that require further study.

1. For prevalence data, most of the studies were of low quality, with very little attempt at random sampling. Hence, the data carry a risk of being biased. Further, some of the studies were done among patients with liver disease; data from these studies have very little relevance for epidemiology.

**Author response:** We agree and we have acknowledged this in the results section and figure 3: *“17/23 studies were cross sectional, reporting HBV population prevalence at a single time point only. Most cohort sampling methods were non-randomised and only 4/21 studies detailed their sample size calculation”.* We have also raised this in the Limitations section: *“The overall quality of studies was low, with non-random sample selection common, no calculation of sample size in most studies and nearly all studies being cross sectional representing only a snapshot of HBV prevalence”.*

Two studies used RPHA technique, which is known to be fairly unreliable.

**Author response:** We agree and have acknowledged this with references in lines 249-251 of the results section *“Two studies used reverse passive haemagglutination for diagnosis of CHB, a method previously demonstrated to have poor sensitivity (28,29,49) (Table 3).”* We have also added to the discussion: *“2/3 studies in this [high risk] group used RPHA for HBsAg detection which is less sensitive than HBsAg, and therefore may underestimate true prevalence of HBV infection*”. Detection method for HBsAg is also included in Table 2. This is important to report as it demonstrates the lack of available accurate screening methods for CHB in many areas of Kenya and highlights potential caveats in the dataset.

The studies in patients with liver disease (the so-called very high risk subgroup) need to be excluded; this will also remove studies that were based on RPHA.

**Author response:** Many people do not find out they have HBV infection until presenting to hospital with jaundice or hepatitis. While the prevalence in this group evidently cannot be extrapolated to the general population, it is nevertheless an important observation that HBV in this setting accounts for such a high proportion of end-stage liver disease. Therefore, it is important and justifiable to present data on HBV prevalence in these populations as well as more generally. For this reason however we did not undertake pooled prevalence analysis of all groups together, but only pooled each individual risk group to avoid a skewed result. ⅔ of the studies in the very-high risk group used RPHA which is known to be unreliable (lines 327-328) – but if anything, this means the prevalence of HBV in people presenting with liver disease is even higher than the estimates we have presented. We have added all these points to the first paragraph of the discussion.

Further, instead of dividing the remaining studies into low-, moderate- and high-risk populations, it may be better to divide these into (i) studies likely to represent general population (the current low risk group), (ii) people living with HIV (the current moderate risk group), (iii) IVDUs and (iv) MSM (the last 2 are currently in high-risk group). This will do away with the artificial risk-based classification, and also account for the results which showed similar positivity rates in the medium and the high risk subgroups.

**Author response:**

We understand the hesitancy of putting people in artificial risk groups. We have added a sentence acknowledging this in the methods and that this is not an externally validated stratification system, however we felt this was a pragmatic approach to a highly heterogenous literature, and made for ease of reference throughout the paper. For the reasons above, we have elected to keep the ‘very high-risk’ classification, but refined by the additional discussion around this group which makes it clear that the data from this group need to be regarded independently from the other classes.

It may also be useful to consider including only those studies that have quality score above a particular cut-off.

**Author response:** We did consider taking this approach during the study analysis. However, only including studies of a certain quality would significantly reduce the available data, and thus restrict the view of what has been reported to date. One of the key findings from this review is the lack of good quality data - describing this in detail is helpful to understand what the shortfalls in data are. We have added a sentence to this end in the limitations section.

2. The Forest plots show the results of both common effects and random effects models. The authors need to choose one (the random effects model is more appropriate) and show the results of only that.

**Author response:** This has been changed to only represent the random effects model.

3. For the data on genotype, the authors start with 9 studies, and then exclude some that included no data (reference 39) and two that had data only for drug resistance mutations (references 46, 47). The above 3 studies should have not even been included in the first place for this part of the analysis. It is unclear from Table 3 or text as to which were the remaining 6 studies that had data on genotype and their sample sizes for this purpose. This needs to be clarified using a separate table for data for this part of the analysis.

**Author Response:** Apologies if this was confusing. We excluded only reference 39 (now reference 25) from the genetic data analysis, as although it reported some sequencing data, there was insufficient detail for us to carry this study forward for further analysis. However, it is important to acknowledge the genetic information presented in this paper. We agree that additional clarity is needed to explain why it was excluded.

References 46 and 47 (now references 23 and 24) were mentioned specifically as they weren’t included in the HBV prevalence section as they only included HBsAg positive patients. We did however include these in the sequencing section as they contained significant information on HBV sequences, leaving 8 studies in total (only one was excluded rather than 3). We have added clarity to the text and have also amended figure 1 (flowchart) to explain studies included/excluded. We have also highlighted in table 2 which studies were used for the genetic information.

4. The paper goes into some other aspects somewhat peripherally, e.g. anti-HBc prevalence, anti-HBs prevalence, HBeAg prevalence, drug resistance mutations, eligibility for treatment, etc. These should not be a part of the paper. Thus, Tables 1 and 7 can be deleted.

**Author response:** We have removed aspects reporting other biomarkers and treatment eligibility data as we agree this was not the primary aim and may distract from the overall message. We have kept information about drug resistance mutations as this is an important point related to available sequencing data and how this information is being used - it also highlights the little information available regarding drug resistance mutations, and the need for more detailed sequencing analysis of HBV in Kenya.

5. The paper is too long and can be shortened.

**Author response:** We agree and have tackled this by removing the sections on other biomarkers and treatment stratification which shortens the paper significantly and makes the message more succinct.
